# Reports and Statistics of the German Hospitals for the Insane

**Published:** 1848-04-01

**Authors:** 


### REPORTS AND STATISTICS OF THE GERMAN HOSPITALS AND ASYLUMS FOR THE INSANE.

Hospital at Hofheim—(from Dr. Amelung's Report.)—200 males and 157 females were in the hospital at the close of the year 1844. During the year 1845, 29 males and 26 females were admitted, and 36 males and 28 females discharged. Of these, 16 were cured, 6 relieved, and 39 died—namely, 23 men and 16 women. This gives a general mortality of about 1 in 10, or $9\frac{1}{2}$ per cent., nearly; the mortality was greater among the males than the females, being 10·4 per cent. of the former, or 1 in 9·9 and 8·7 per cent. of the latter, or 1 in 11·4. Of the 55 persons admitted, 23 males and 23 females were insane; 3 males and 3 females imbecile or idiotic, 1 (male) was epileptic, and 2 males paralytic: 14 of the admissions had been in the hospital before. The ages of the 55 were—

| | Males. | Females. | Total. |
|---|---|---|---|
| Aged from 10 to 20 | 5 | 0 | 5 |
| „ 20 to 30 | 11 | 8 | 19 |
| „ 30 to 40 | 1 | 6 | 7 |
| „ 40 to 50 | 8 | 8 | 16 |
| „ 50 to 60 | 3 | 3 | 6 |
| „ 60 to 70 | 1 | 1 | 2 |

As regards their domestic condition, 19 males and 12 females were unmarried; 6 males and 7 females married; 4 were widowers and 7 widows. The admissions were most numerous in April, (8,) May, (8,) and September, (9.)

Moral and physical causes were generally combined; but in 18 males and 13 females, they were principally physical, and in 11 males and 13 females, principally moral. In 4 males and 10 females, an hereditary tendency to the disease was observed.

Only 22 presented any hopes of restoration; 3 males and 5 females were dismissed cured during the year; 9 males and 5 females were improved, and 3 males and 2 females died. Of those who were cured or relieved, the duration of insanity previously to admission was as follows:—

| | Males. | Females. | Total. |
|---|---|---|---|
| Affected from 1 to 3 months | 4 | 4 | 8 |
| „ 3 to 6 „ | 2 | 0 | 2 |
| „ 6 to 8 „ | 1 | 2 | 3 |
| „ 9 to 12 „ | 1 | 2 | 3 |
| „ 1 to 2 years | 2 | 0 | 2 |
| „ several years | 2 | 2 | 4 |
| | 12 | 10 | 22 |

During the year, 111 persons were under special medical treatment—namely, 50 males and 61 females. The cerebral diseases for which they were treated were as follow:—

| | Males. | | Females. |
|---|---|---|---|
| Acute insanity ... ... ... ... | 6 | ... | 3 |
| Chronic „ ... ... ... ... | 25 | ... | 20 |
| Periodic „ ... ... ... ... | 9 | ... | 15 |
| Melancholy ... ... ... ... | 0 | ... | 4 |
| Idiocy ... ... ... ... | 6 | ... | 9 |
| Insanity, with epilepsy ... .. | 2 | ... | 7 |
| Uncomplicated epilepsy ... ... | 2 | ... | 3 |
| Total ... | 50 | | 61 |

The proportion of deaths was unusually great; the cause, principally chronic disease of the lungs and nervoûs system. The number of attendants is 17, being a proportion of 1 to 23·4 patients.

With regard to the treatment pursued at Hofheim, Dr. Amelung reports that out-door employments for the men, and ordinary female occupations for the women, were the most efficient adjuvants. The patients have a library and newspapers; cards, chessmen, &c., for various games; a singing-club, and an instrumental band, consisting of several violins, a violoncello, clarionet, and piccolo. On Christmas eve they had a Christmas-tree, and various little presents; and on the 28th of December, a ball, at which the house-band performed. These festivities were conducted with the greatest decorum, and gave great satisfaction.

The means of restraint used at Hofheim are, rooms built for the purpose, the strait-waistcoat, chair, and girdle, or belt. The chair was seldom used, except to administer medicines, or apply remedies sometimes; or as a punishment. When used as a means of restraint, it was not always annoying. One very violent patient, who could not be restrained by any other means, often asked for it as her "confessional," and another was often indulged, at her special request, with a seat in it. No suicide or other accident took place during the year.

The cost of medicines was 908 florins, nearly, or not quite 3*s.* 8*d.* per head.—*Allgemeine Zeitschrift für Psychiatrie*, vol. iv. part 1.

Hospital at Eberbach, in the Rheingau, for the Grand Duchy of Nassau.—(Report by Dr. Lindpainter.)—At the close of 1843, the hospital contained 137 patients; 24 were admitted and 24 discharged during 1844. Of the 24 admitted, 8 males and 4 females were discharged, apparently cured; 4 males remained unrelieved, and 4 males and 4 females died, or 1 in 3 of the admissions! Of 40 patients discharged during the previous three years, 21 remained well, 12 showed a tendency to insanity, 6 soon suffered relapses, and 1 died.

In 161 cases, the disease depended, in 42 males and 43 females, upon physical causes; in 54 males and 21 females, upon moral causes. Amongst the latter, anxiety about a livelihood and misfortunes were ascertained to be the causes, in 14 cases; onanism, in 12 cases, (males;) and drunkenness, in 13 cases, also males. In 25, the predisposition to the disease was hereditary; in 14, there was congenital idiocy; in 9, a complication, with epilepsy.

During the winter, from 20 to 24 patients learnt writing and accounts. The medicines cost 5*s.* per head. Reckoning the dollar at three shillings, patients of the second class averaged a cost of 27*l.* 9*s.* per annum; of the third class, 23*l* 11*s.*; of the fourth class, the males averaged an annual cost of 18*l.* 15*s.*, and the females of 17*l.* 5*s.*—*Allgemeine Zeitschrift für Pyschiatrie*, &c., vol. iv. part 1.

Hospital at Sorau.—(Report by Dr. Schnieber, Physician to the Hospital.)—This Report contains few statistical details, and is principally taken up with an exposition of the plan of treatment followed, and with details of cases. At the close of the year 1843, the hospital contained 138 patients—78 males and 60 females. During the year 1844, the admissions were, 37 males and 14 females; 8 males and 5 females were dismissed cured, and 12 males and 4 females died, or 1 in 9·6 of the former, and 1 in 18·5 of the latter. This is a fearful discrepancy. The various forms of mental disorder were thus distributed:—

| | Males. | | Females. |
|---|---|---|---|
| Permanent insanity and mania ... | 48 | ... | 38 |
| Periodic ... ... ... ... | 2 | ... | 1 |
| Uncomplicated imbecility ... ... | 25 | ... | 18 |
| Imbecility, with maniacal paroxysms | 7 | ... | 4 |
| ,, epilepsy ... ... | 4 | ... | 0 |
| Epilepsy, with insanity or idiocy, and maniacal paroxysms | 8 | ... | 2 |

The hospital at Sorau is not simply an hospital for the cure of insanity, but receives incurable in especial, including all kinds of mismanaged cases. Every requisite means for the employment of all such patients as are in the least degree capable of labour, is provided. The higher and educated classes are occupied partly in sawing wood and gardening, partly in reading, mathematics, translations of works, transcribing, chess, &c. Those of a lower class, with all kinds of house and field work. Persons of a trade are employed, if possible, at their trade. The tailor and shoemaker are fully employed, and work is found for the whitesmith, joiner, turner, and clock and watch maker. The dressing of feathers is found to be a simple and an easy employment. Dr. Schnieber observes, that great patience and perseverance will succeed in inducing the most apathetic and listless to engage in active occupation. Various gymnastics are also provided.

As to the means of restraint used, and the introduction of the non-restraint system, Dr. Schnieber observes that it is not possible to abandon the former altogether, or to adopt the latter without a larger number of educated and skilful attendants. At Sorau, the attendants are principally invalided soldiers, or persons of the lower class, whose wages are small; the male attendants having only twelve shillings per month, and board, and the females less. He has often experienced the disadvantage of having uninstructed or harsh attendants, whether in curing or alleviating insanity. That the attendants may know their duty, distinctly written instructions are read over to them several times in a year, and each new comer receives special directions. If these instructions are disobeyed, the attendants are liable to dismissal and further punishment.

The means of restraint used at Sorau are the ordinary strait-waistcoat, mufflers, and belts. A sort of mask, like a bee-hive in form, is provided for those who put everything they get into their mouth, or who, when their hands are restrained, tear and destroy with their teeth. Palisaded rooms are also in use, and, in particular cases, the revolving chair. This is adapted to the most intractable cases, when all other means fail, and the fear of it is so great, that the use of it from three to five minutes tames the most disobedient and unruly.

Prayers, with singing, are conducted morning and evening, and on Sundays, service is performed in the church, where there is an organ and a stove. It is rare that the patients cause the slightest disturbance.

Seven fully detailed cases and diet-tables accompany the Report.—Ibid. p. 74, *seq.*

Statistics of the Institution for the Insane at Marsberg, for 1845.—This institution comprises two distinct departments—namely, an asylum for the safe custody and care of the patients who may be incurable, and an hospital for the curable. There is also an asylum for patients of the higher class, and a private institution belonging to the director. We take the totals of the patients in all these departments. At the end of the year 1844, there were 168 males and 127 females. During the year 1845 were admitted 79 males and 53 females; 69 males and 43 females were discharged, leaving a total during the year of 178 males and 137 females; the daily average of inmates was 310—namely, 178 males, 132 females. The dismissals were as follow:—

| | Cured. | Uncured. | Relieved. | Died. |
|---|---|---|---|---|
| Males ... | 29 ... | 9 ... | 2 ... | 17 |
| Females ... | 13 ... | 3 ... | 3 ... | 10 |

The rate of mortality of the males was therefore 9·5 per cent., and of the females 7·6 per cent. The patients averaged by their labour the sum of 8*s.* 4*d.* per head; they cost for food per head, 6*l.* 16*s.* 5*d.*; clothing, 16*s.* 6*d.*; fire, candle, washing, attendance, and sundries, 8*l.* 10*s.* 6*d.*; and deducting their earnings, the average annual cost of each patient was 15*l.* 14*s.* In making these estimates we have not considered fractions of the silbergroschen.

Statistics of Insanity in Norway. By Professor Holsh.—(Zeitschrift für Psychiatrie, vol. iv. part 3. Translated from the second volume of the Norwegian Magazine of Medicine 1846.)—The statistics of insanity in Norway have been twice officially taken, in conjunction with the general census: first in 1825, and then in 1835. The returns were made under the four heads of mania, melancholia, (monomania,) dementia, and idiotia. As the census returns were made in the country by the parochial clergy, village schoolmasters, and other persons incompetent to establish a diagnosis in the respective instances of which they made a return, no great degree of accuracy as to the different forms of insanity was to be expected. The statistics must, then, be received on this point with some doubt as to their correctness.

The kingdom of Norway presented the following proportions of insane persons at the two periods named, to the general population. We omit fractions:—

| | 1835. | | | 1825–6. | | |
|---|---|---|---|---|---|---|
| | Males. | Females. | Total. | Males. | Females. | Total. |
| In the towns ... | 1 to 349 | 1 to 406 | 1 to 377 | 1 to 449 | 1 to 553 | 1 to 498 |
| In the country ... | 1 to 320 | 1 to 339 | 1 to 329 | 1 to 516 | 1 to 603 | 1 to 557 |
| In the whole kingdom | 1 to 322 | 1 to 345 | 1 to 334 | 1 to 508 | 1 to 597 | 1 to 550 |

This table presents a frightful increase in the number of insane persons in the ten years from 1825 to 1835. Professor Holst can only attribute it to a great increase of intemperance, consequent upon an abolition of the duty on spirits. The increase, allowing for the increase of population, was in the towns to an amount equalling 32·9 per cent.; in the rural districts, 69·0 per cent.; for the whole kingdom, 64·8 per cent.; the increase in the population bearing no relation to these figures. The increase in the various forms of insanity was, mania, 41 per cent.; melancholia, 69 per cent.; dementia, 52 per cent.; and idiocy (congenital), 150 per cent., principally in the rural districts! This is a frightful illustration of the ravages and cost of intemperance.

The following are the numbers of each class of mental disease for the whole kingdom :—

| | 1835. | | | 1825–6. | | |
|---|---|---|---|---|---|---|
| | Males. | Females. | Total. | Males. | Females. | Total. |
| Maniacal ... ... | 363 | 360 | 723 | 270 | 242 | 512 |
| Melancholic ... | 304 | 331 | 635 | 198 | 178 | 376 |
| Imbecile ... ... | 261 | 259 | 520 | 168 | 173 | 341 |
| Idiotic ... ... | 845 | 813 | 1698 | 369 | 311 | 680 |
| Total ... | 1813 | 1763 | 3576 | 1015 | 904 | 1909 |

We subjoin a table, (constructed by ourselves from the data of Professor Holst,) showing the relative prevalence in the civic and rural populations of Norway of the different forms of insanity at the period of the census in 1835.

| Form of Insanity. | Civic Populations. | | Rural Populations. | |
|---|---|---|---|---|
| | Males, 1 to | Females, 1 to | Males, 1 to | Females, 1 to |
| Mania ... ... | 1078·2 | 1107·2 | 1712·1 | 1868·6 |
| Melancholia ... | 1755·9 | 1500·9 | 1947·6 | 1894·7 |
| Dementia ... ... | 1755·9 | 2597·8 | 2318·2 | 2325·7 |
| Idiotia ... ... | 1254·3 | 1984·0 | 525·5 | 695·6 |

It is worthy of notice, that in all the forms of insanity, in both classes of population, the men are more liable than the women, with the exception of melancholia, in which, in both classes, there is a greater proportion of women affected. The immense preponderance of idiocy in the rural districts can only, we think, arise from immigrant idiots. The inhabitants of the towns, it seems to us, would be apt to get their idiot relatives as much out of sight as possible, by sending them into the country.

This paper contains also the statistics of blindness and deaf-dumbness; and although not exactly pertinent to our subject, we subjoin a short abstract, feeling assured it will interest our readers.

The total number of persons blind of one eye in Norway, in 1835, was 4044; of these, 2435 were males, and 1609 females. The total number blind of both eyes was 2109; of these, 1028 were males, 1081 females. They were distributed in the rural and civic districts as per subjoined table:—

| Nature of Population. | Blind of one Eye. | | | Blind of both Eyes. | | |
|---|---|---|---|---|---|---|
| | Males, 1 to | Females, 1 to | Both Sexes, 1 to | Males, 1 to | Females, 1 to | Both Sexes, 1 to |
| Civic population ... | 574·4 | 734·2 | 648·3 | 787·9 | 833·9 | 811·3 |
| Rural population ... | 225·0 | 357·2 | 277·2 | 551·5 | 541·9 | 546·6 |
| Population of the whole kingdom | 240·4 | 378·8 | 295·4 | 569·4 | 563·8 | 566·5 |

It will be seen that the proportion of persons blind of one eye is more than twice as great in the country as in the towns, and of those blind of both eyes, about one third greater. Professor Holtz attributes this disparity to the greater want of medical aid in the provinces. In both classes of population the males preponderate greatly over the females.

There were 1081 persons deaf and dumb in Norway in 1835—namely, 588 males, and 493 females. They were distributed as follow:—

Distribution of the Deaf and Dumb in Norway.

| Nature of Population. | Males, 1 to | Females, 1 to | Both Sexes, 1 to |
|---|---|---|---|
| Civic population ... | 1059·6 | 1570·8 | 1277·2 |
| Rural population ... | 970·2 | 1204·2 | 1076·6 |
| General population ... | 978·9 | 1236·2 | 1095·2 |

It has been long known that a greater number of males are deaf and dumb than of females; these statistics confirm this general opinion,—there were 27 per cent. more males in Norway.

Statistics of Suicide in the Grand Duchy of Baden.—(Zeitschrift für Psychiatrie, vol. iv. part 1.)—The subjoined table is an official document issued by the minister of justice. It is remarkable that of the 126 cases of suicide or attempted suicide, 65 occurred during the months of May, June, July, and August, and 61 during the remaining eight months. Ten persons (males) attempted suicide; three were aged from 18 to 25; two, from 25 to 35; four, from 35 to 45; and one, 45 to 50.

TABLE OF THE MODES OF DEATH ADOPTED BY 116 SUICIDES IN THE GRAND DUCHY OF BADEN.—MALES, 91 ; FEMALES, 25.

| Age. | Poison. | | Hanging. | | Shooting. | | Drowning. | | Cut Throat. | | Suffocation. | | Precipitation from a height. | |
|---|---|---|---|---|---|---|---|---|---|---|---|---|---|---|
| | M. | F. | M. | F. | M. | F. | M. | F. | M. | F. | M. | F. | M. | F. |
| Under 18 years | ... | ... | 1 | ... | ... | ... | 1 | | | | | | | |
| 18 to 25 „ | ... | ... | 9 | 2 | 9 | 1 | 2 | 4 | ... | ... | 1 | | | |
| 25 to 30 „ | 2 | ... | 5 | 1 | 1 | 1 | 1 | 1 | | | | | | |
| 30 to 35 „ | ... | ... | 3 | ... | 3 | ... | 1 | 1 | | | | | | |
| 35 to 40 „ | ... | ... | 4 | 1 | 2 | ... | 3 | ... | 1 | ... | 1 | | | |
| 40 to 45 „ | ... | ... | 12 | 3 | 1 | ... | 3 | 1 | 2 | ... | ... | ... | ... | 1 |
| 45 to 50 „ | ... | ... | 9 | 3 | 2 | ... | 3 | 1 | 1 | ... | ... | ... | ... | 2 |
| 50 to 55 „ | ... | ... | 5 | 1 | 1 | | | | | | | | | |
| 70 & unknown | ... | ... | 2 | ... | ... | ... | ... | 1 | | | | | | |
| Total ... | 2 | ... | 50 | 11 | 19 | 2 | 14 | 9 | 4 | ... | 2 | ... | ... | 3 |

TABLE OF THE MOTIVES FOR SUICIDE IN 125 CASES IN THE GRAND DUCHY OF BADEN.

